# Acclimatisation with Inhalation Sedation in Paediatric Dentistry: A Scoping Review

**DOI:** 10.3390/dj14100623

**Published:** 2026-09-30

**Authors:** Evan J. Bowers, Christopher C. Donnell

**Affiliations:** 1 Department of Paediatric and Special Care Dentistry, Hull City Health Care Partnership CIC, Hull HU3 4AE, UK; 2 Department of Paediatric Dentistry, Charles Clifford Dental Hospital, Sheffield Teaching Hospitals NHS Foundation Trust, Sheffield S10 2SZ, UK; 3 Academic Unit of Oral Health, Dentistry and Society, School of Clinical Dentistry, University of Sheffield, Sheffield S10 2TA, UK

**Keywords:** paediatric dentistry, inhalation sedation, nitrous oxide, acclimatisation, familiarisation, dental anxiety, behaviour management, scoping review

## Abstract

**Background:** Acclimatisation with inhalation sedation may involve providing minimally invasive dental treatment alongside a graduated introduction to inhalation sedation, its equipment, and the wider sensory experience. These preparatory visits may help assess a child’s suitability for subsequent treatment under inhalation sedation while supporting behavioural familiarisation with the sedation environment. However, the extent to which acclimatisation has been clearly characterised or formally evaluated remains uncertain. **Aim:** To explore and map the literature relating to acclimatisation with inhalation sedation in paediatric dentistry. **Methods:** A scoping review was conducted in accordance with Joanna Briggs Institute guidance and reported in line with PRISMA-ScR recommendations. MEDLINE, Embase, Cochrane Library, Scopus and Web of Science were searched alongside grey literature sources and citation searching. Reports involving staged or introductory exposure to inhalation sedation prior to definitive paediatric dental treatment and available in English were eligible for inclusion. Data were extracted and checked by both reviewers, and the findings synthesised narratively. **Results:** Four reports representing three distinct studies met the inclusion criteria, comprising two protocols describing the same planned trial and two completed studies involving 56 and 20 children. Terminology, interventions and outcome measures varied considerably. One study reported increased initial familiarisation time without fewer visits or shorter overall treatment, while the other found no significant between-group difference in dental anxiety. The protocol reports provided no outcome data. **Conclusions:** Acclimatisation with inhalation sedation has been described in clinical guidance and a limited number of reports, but remains inconsistently defined and insufficiently evaluated. Current evidence does not establish its clinical effectiveness. Clearer definitions, standardised outcome measures and adequately powered prospective studies are required.

## 1. Introduction

Dental anxiety and behavioural management challenges remain common barriers to the successful delivery of paediatric dental care. Contemporary paediatric dentistry approaches emphasise the importance of non-pharmacological behavioural management as the foundation of care, with pharmacological techniques utilised adjunctively where appropriate [1,2,3,4]. Inhalation sedation (IHS), also termed ‘Relative Analgesia (RA)’, using nitrous oxide and oxygen is a well-established and effective adjunct within paediatric dentistry, with reported success rates generally high among appropriately selected children [5,6,7]. However, successful inhalation sedation depends on factors extending beyond pharmacology alone, including communication, behavioural cooperation, tolerance of the nasal hood and wider patient management approaches [8,9].

Current sedation guidance acknowledges that a brief “trial” of nitrous oxide/oxygen during assessment appointments may be helpful for the psychological preparation of some children [3]. However, relatively limited detail is provided regarding which children may benefit most from acclimatisation, how such interventions should be structured, or what outcomes acclimatisation is intended to achieve within paediatric dental care pathways.

Consequently, some clinicians utilise acclimatisation appointments prior to definitive treatment under inhalation sedation. These appointments are distinct from routine sedation assessment and subsequent definitive treatment under IHS, and may involve behavioural or environmental familiarisation through graded exposure, pharmacological exposure to nitrous oxide/oxygen, or both [10]. However, the extent to which these concepts have been defined or formally evaluated in the literature remains unclear [11,12]. Broader multidisciplinary approaches involving behavioural support and confidence building have also been described within salaried and community dental services [13].

Service pressures, including general anaesthesia waiting lists, workforce training and the environmental impact of nitrous oxide use, provide additional context but remain secondary to establishing the clinical purpose and evidence base for acclimatisation [14,15,16]. Despite descriptions of acclimatisation within paediatric dentistry, there remains a paucity of literature examining how it is defined, implemented and evaluated. This review did not seek to reassess the established efficacy or safety of nitrous oxide/oxygen sedation itself, but to examine the distinct preparatory encounter that may precede definitive treatment. Therefore, the aim of this scoping review was to explore and map the existing literature relating to acclimatisation with inhalation sedation in paediatric dentistry.

## 2. Materials and Methods

### 2.1. Study Design

Given the anticipated heterogeneity of the available evidence, alongside the exploratory nature of the review question, a scoping review methodology was selected. Scoping reviews are particularly suited to areas where the literature remains sparse, poorly defined, or methodologically diverse, and allow for preliminary mapping of emerging evidence and identification of knowledge gaps [17]. The review was conducted in accordance with the methodological framework proposed by the Joanna Briggs Institute (JBI) for systematic scoping reviews [18,19], and reported in line with the Preferred Reporting Items for Systematic Reviews and Meta-Analyses extension for Scoping Reviews (PRISMA-ScR) checklist [20]. The review was guided by a protocol developed before searching and screening, although it was not prospectively registered or published.

### 2.2. Review Question

The review sought to explore how acclimatisation with inhalation sedation has been conceptualised, operationalised and evaluated within paediatric dentistry. The review question was:

*“How has acclimatisation with inhalation sedation prior to definitive dental treatment been described and evaluated in children and adolescents undergoing paediatric dental care?”*

For this review, acclimatisation was defined as a planned preparatory appointment before definitive treatment under IHS. This could involve behavioural or environmental familiarisation with the nasal hood and sedation equipment, with or without administration of nitrous oxide/oxygen. Visits described as acclimatisation, familiarisation, preparatory, introductory or “trial run” were included. General behavioural preparation without an explicit link to subsequent IHS was excluded.

Children and adolescents were defined as individuals aged 18 years and under.

### 2.3. Search Strategy

The literature search strategy was developed in consultation with a healthcare librarian. MEDLINE, Embase, Cochrane Library, Scopus and Web of Science were searched, alongside Google Scholar, grey literature sources and reference-list screening. Controlled vocabulary and free-text terms relating to inhalation sedation, nitrous oxide, acclimatisation and related preparatory concepts, behaviour management and paediatric dentistry were combined. The full Ovid MEDLINE strategy is presented in Box 1, with strategies for the remaining databases provided in Supplementary File S1. OpenGrey, EThOS, Nexis and ProQuest were searched using keyword combinations based on the Scopus strategy and were adapted to the functionality of each platform. Google and Google Scholar were searched using simplified combinations of the core concepts, and the reference lists of included reports and other relevant sources were screened for additional records.

Searches included literature published from database inception to May 2026. No restriction was placed on study design due to the expected paucity of evidence.

Box 1 **Search strategy for Ovid MEDLINE.**

1  (inhalation sedation or relative analgesia or “gas and air” or happy air or laughing gas or (nitrous oxide and (sedation or (anaesthe* or anesthe*)))).tw.

2  exp dentistry/or exp dental care/

3  (dentist* or dental).mp. [mp = title, book title, abstract, original title, name of substance word, subject heading word, floating sub-heading word, keyword heading word, organism supplementary concept word, protocol supplementary concept word, rare disease supplementary concept word, unique identifier, synonyms, population supplementary concept word, anatomy supplementary concept word]

4  2 or 3

5  1 and 4

6  limit 1 to dentistry journals

7  5 or 6

8  exp Acclimatization/or acclimati?ation.mp. or acclimation.mp.

9  systematic desensiti?ation.mp.

10  exp Cognitive Behavioral Therapy/or cbt.mp. or cognitive behavior* therapy.mp. or cognitive behaviour* therapy.mp.

11  graded exposure.mp.

12  exp Behavior Therapy/

13  (introduc* or acceptance).ti.

14  (familiari?ation or (prepar* adj2 appointment*) or (trial adj1 run*)).mp.

15  8 or 9 or 10 or 11 or 12 or 13 or 14

16  7 and 15

17  exp Child/or exp Pediatric Dentistry/or exp Child, Preschool/or exp “Dental Care for Children”/or exp Adolescent/

18  (pediatric or paediatric or child or children).mp.

19  17 or 18

20  16 and 19

21  limit 16 to “all child (0 to 18 years)”

22  20 or 21

### 2.4. Eligibility Criteria

Studies were considered eligible if they:

- Involved children or adolescents aged 18 years or under;
- Related to inhalation sedation or nitrous oxide sedation within paediatric dentistry;
- Described or evaluated a staged introductory, preparatory or acclimatisation appointment explicitly linked to planned definitive treatment under IHS, involving exposure to the sedation equipment, nitrous oxide/oxygen, or both;
- Were available in the English language.

Given the exploratory aims of the review, the study protocol allowed for a range of evidence sources, including clinical studies, theses, protocols and the grey literature.

Studies were excluded if they:

- Focused exclusively on inhalation sedation efficacy without any acclimatisation component;
- Related solely to non-pharmacological preparation for dental treatment;
- Concerned adult populations only;
- Were not available in English;
- Consisted solely of abstracts without accessible full text.

Protocol publications were eligible because they described how acclimatisation interventions had been conceptualised and structured. They were treated as descriptions of planned studies and not as evidence of clinical effectiveness.

### 2.5. Study Selection

Records identified through database and grey literature searching were imported and screened manually by EJB and CCD following removal of duplicates. Titles and abstracts were initially screened for relevance, followed by full-text review where appropriate. Screening was performed independently by both reviewers.

### 2.6. Data Extraction

Data were extracted using a structured data-charting form developed specifically for the review. The form was piloted by both reviewers before data extraction. Each reviewer extracted part of the dataset, and all entries were cross-checked by the other reviewer. Any uncertainties or discrepancies were resolved through discussion and consensus. Extracted variables included study characteristics, participant demographics, intervention design, outcome measures, and key findings relevant to the review question. Particular attention was paid to how acclimatisation was defined and implemented within each study. The full data extraction fields are presented in Box 2.

Box 2 **Data extraction fields.**

Author(s)

Year of publication

Title

Journal/source

Study aims/purpose

Country of origin

Study design

Study population/sample size

Intervention and comparator details

Outcome measures

Key findings

Relevance to the review question

### 2.7. Data Synthesis

Given the substantial heterogeneity anticipated between studies in intervention design, terminology, outcome measures, and methodological approach, meta-analysis was not considered appropriate. Instead, extracted data were compared across reports, grouped at study level where appropriate, and organised into descriptive categories covering terminology and conceptual framing, intervention structure and delivery, and reported outcome measures. These categories were developed iteratively from the charted data and agreed by both reviewers through discussion. This process supported a structured narrative synthesis rather than formal thematic analysis.

## 3. Results

### 3.1. Overview of Included Literature

**Figure 1:**
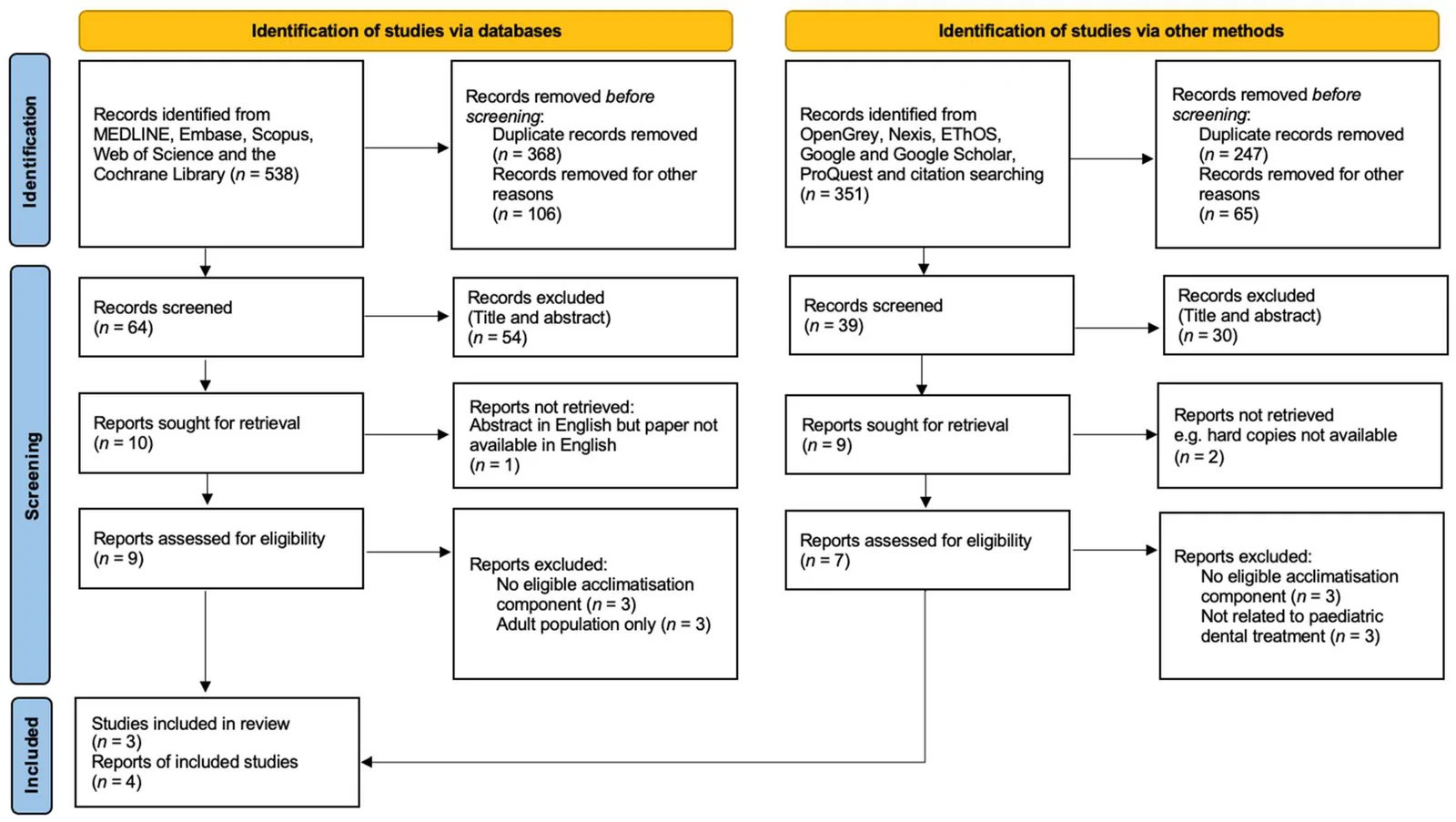
PRISMA flow diagram.

The database and grey literature searches identified 889 records (Figure 1). Before screening, 615 duplicate records were removed, together with a further 171 records that were not available in English or were ineligible publication types, including editorials, correspondence, corrections and conference abstracts. Of the remaining 103 records, 84 were excluded following title and abstract review. Nineteen reports were sought for retrieval; three could not be retrieved, leaving 16 reports for full-text assessment. Twelve reports were excluded, resulting in four included reports representing three distinct studies. Two reports were protocols describing the same planned UAE trial; the other two reported completed empirical studies, comprising one unpublished postgraduate thesis and one published clinical study. The reports originated from the United Kingdom (UK), United Arab Emirates (UAE) and the Netherlands, and were published between 1991 and 2022 [21,22,23,24].

Considerable variation was evident in study design, terminology, intervention content and outcome measures. Planned or delivered approaches ranged from familiarisation with the nasal hood and sedation equipment to exposure to nitrous oxide/oxygen and broader behavioural preparation. The two protocol reports presented planned outcomes only and provided no effectiveness data. The two completed studies assessed different outcomes and were therefore unsuitable for direct comparison or quantitative synthesis. Characteristics of the included reports are summarised in Table 1.

### 3.2. Narrative Synthesis

#### 3.2.1. Terminology and Conceptual Framing

Terminology and conceptual framing varied across the four reports, with terms such as “acclimatisation”, “familiarisation”, “introductory visit”, “preparatory appointment” and “trial run” used inconsistently. The two UAE protocol reports described the same planned trial and used acclimatisation to refer to brief nitrous oxide/oxygen exposure before operative treatment. Palikaraki compared nitrous oxide/oxygen exposure during an assessment visit with mask familiarisation using oxygen alone. Veerkamp et al. did not define acclimatisation as a separate intervention; both groups received introductory behavioural procedures, with one group additionally receiving nitrous oxide during treatment [21,22,23,24].

The reports therefore addressed related but distinct combinations of pharmacological exposure, equipment familiarisation and behavioural preparation. They should not be interpreted as evaluating a single standardised intervention.

#### 3.2.2. Structure and Delivery of Acclimatisation Appointments

The two UAE protocol reports described the same planned trial rather than separate interventions. Children allocated to acclimatisation were to receive approximately 15 min of nitrous oxide/oxygen during a preparatory visit before operative treatment; the comparator involved an explanation of IHS without practical exposure [21,22].

In Palikaraki’s completed study, 20 children were allocated to nitrous oxide/oxygen exposure (*n* = 11) or oxygen-only mask familiarisation (*n* = 9) during an assessment visit, when both groups received a prophylaxis. Subsequent treatment was undertaken under IHS in both groups. Veerkamp and colleagues randomised 56 fearful children to behavioural management alone or behavioural management with nitrous oxide during treatment. Both groups underwent introductory procedures, so the comparison did not isolate a separate acclimatisation appointment [23,24].

These approaches differed in timing, pharmacological exposure, and whether preparation occurred during a separate visit or was embedded within treatment.

#### 3.2.3. Evaluation and Outcome Measures

The two UAE protocol reports proposed behavioural measures, anxiety scores, treatment completion, salivary alpha-amylase and cortisol, and real-time physiological monitoring. Neither report provided outcome data [21,22].

In Palikaraki’s study, the mean reduction in MCDASf score from baseline to the end of treatment was 2.55 (SD 8.02) following nitrous oxide/oxygen exposure and 7.22 (SD 5.12) following oxygen-only mask familiarisation. The between-group difference in change was not statistically significant (mean difference −4.68; *p* = 0.148). Treatment was completed by 10 of 11 children in the nitrous oxide/oxygen group and all 9 children in the oxygen-only group (*p* = 1.000). No statistically significant between-group differences were identified in the reported behavioural or physiological measures. Recruitment was disrupted by the COVID-19 pandemic and the study was underpowered [23].

In Veerkamp and colleagues’ study, the mean number of treatment sessions was 4.8 (SD 1.2) with behavioural management alone and 5.2 (SD 1.5) with additional nitrous oxide; this difference was not statistically significant. Initial familiarisation took longer with nitrous oxide for both treating dentists: 20 compared with 36 min (*p* = 0.05) and 24 compared with 41 min (*p* = 0.04). Total treatment time was also longer with nitrous oxide for one dentist (158 compared with 218 min; *p* = 0.02), but the difference was not statistically significant for the other dentist (116 compared with 147 min) [24]. As both groups received introductory behavioural procedures and nitrous oxide was administered during treatment, these findings do not provide an estimate of the effect of a separate preparatory acclimatisation appointment.

The interventions and outcomes differed substantially between the two completed studies, preventing direct comparison or quantitative synthesis.

## 4. Discussion

To the authors’ knowledge, this is the first scoping review specifically exploring acclimatisation with inhalation sedation in paediatric dentistry. Four reports representing three distinct studies were identified [21,22,23,24]. Two reports were protocols describing the same planned trial and contained no results, leaving only two completed datasets: one small postgraduate thesis and one published clinical study that did not evaluate a separate acclimatisation appointment. Consequently, the evidence available to inform clinical effectiveness was extremely limited. Terminology, intervention content, timing and outcomes varied considerably across the reports, encompassing behavioural or equipment familiarisation, pharmacological exposure to nitrous oxide/oxygen, or a combination of these approaches [21,22,23,24]. Acclimatisation cannot therefore be regarded as a single standardised intervention, and the available evidence does not establish whether a preparatory appointment improves anxiety, cooperation, treatment completion or service outcomes.

Terminology was used inconsistently across the included literature. The UAE protocols used “acclimatisation” to describe a separate preparatory visit involving nitrous oxide/oxygen exposure, whereas Palikaraki distinguished this from oxygen-only familiarisation with the nasal hood. Veerkamp et al. embedded introductory behavioural procedures within treatment for both groups and did not evaluate a separate preparatory appointment [21,22,23,24]. These approaches represent distinct combinations of pharmacological exposure, equipment and environmental familiarisation, and behavioural preparation or graded exposure, and should not be treated as interchangeable. Behaviour management and sedation guidance recognise related concepts such as psychological preparation, graded exposure, familiarisation and preparatory assessment, but do not define acclimatisation as a standardised intervention or specify how it should be delivered [10,11,13]. This breadth of practice makes comparison difficult unless the intervention being described is clearly specified.

Palikaraki was the only completed study to directly compare pharmacological exposure to nitrous oxide/oxygen during an assessment visit with oxygen-only mask familiarisation before subsequent treatment under IHS [23]. No statistically significant between-group differences were identified in anxiety, behavioural, physiological, or treatment-completion outcomes. However, only 20 participants were analysed, and the study was underpowered following disruption caused by the COVID-19 pandemic. The findings therefore neither establish equivalence between the approaches nor demonstrate that prior nitrous oxide/oxygen exposure is ineffective. Furthermore, the intervention involved a single brief exposure rather than a broader staged acclimatisation pathway, limiting its applicability to other forms of acclimatisation used in clinical practice.

Sedation guidance from the United Kingdom, Europe and North America emphasises patient selection, anxiety assessment, behavioural support and titration of nitrous oxide/oxygen, but provides limited direction on whether preparatory exposure is indicated or how it should be structured [2,3,25,26]. Tools such as the paediatric Indicator of Sedation Need may support assessment of sedation need, referral and treatment planning, but do not identify which children would benefit from a separate acclimatisation appointment [27,28]. Consequently, the role of acclimatisation between assessment and definitive treatment remains based largely on clinical judgement rather than comparative evidence.

At service level, work concerning outpatient activity coding within English paediatric dental services illustrates the difficulty of recognising acclimatisation as a distinct clinical activity [29,30]. Recognition within a coding framework may document service provision, but does not establish clinical effectiveness or a preferred intervention structure. As coding systems and care pathways vary, this English context should not be assumed to apply internationally.

The included studies did not establish whether acclimatisation reduces the need for general anaesthesia or improves longer term treatment completion. The broader clinical literature nevertheless describes circumstances in which IHS may be used within staged care to facilitate preventive, minimally invasive, or stabilisation treatment before more extensive care, including general anaesthesia [5,6,10]. This provides a potential clinical rationale, but not evidence that an acclimatisation appointment itself produces these outcomes. Avoidance of general anaesthesia should also not necessarily be regarded as the only measure of success, as some children may complete limited care under IHS while still requiring general anaesthesia for other treatment. Treatment tolerance may develop incrementally rather than represent a fixed binary outcome [31]. However, none of the included studies evaluated longer term treatment pathways, general anaesthesia use, waiting times or resource use, and these potential benefits remain unproven.

Any proposed acclimatisation pathway must also be considered alongside clinical time, workforce capacity and patient access. Dental schools in the United Kingdom and Ireland have reported reduced undergraduate exposure to conscious sedation despite continuing clinical demand [16], while newer initiatives have sought to strengthen postgraduate conscious sedation competence [32]. Involvement of the wider dental team, including dental therapists, in confidence building and behavioural support has also been described [13]. These sources provide relevant service context but do not demonstrate the effectiveness of acclimatisation or establish which professionals should deliver it.

The environmental impact of nitrous oxide has received increasing attention within healthcare and dentistry [14,15]. A national quality improvement project in the United Kingdom identified variation in nitrous oxide administration, wastage and the use of acclimatisation procedures, and suggested that acclimatisation involving sedation should be considered for individual patients rather than routinely provided [15]. These findings concern service delivery and emissions reduction, not clinical effectiveness. As this review identified no consistent definition of acclimatisation, its environmental and service consequences cannot be meaningfully compared unless intervention components, including whether nitrous oxide/oxygen is administered and for how long, are clearly reported.

Several limitations should be acknowledged. Only four reports representing three distinct studies were identified, including two protocols describing the same planned trial without outcome data. Of the two completed datasets, one was an unpublished postgraduate thesis involving 20 analysed participants and was underpowered following disruption caused by the COVID-19 pandemic; the other was an older clinical study that did not isolate a separate preparatory acclimatisation appointment. The review was restricted to reports available in English, and relevant evidence may remain unpublished or embedded within local clinical practice. Dedicated clinical trial registries were not searched, which may have resulted in the omission of ongoing or unpublished studies. Although the review protocol was developed before searching and screening commenced, it was not registered or published. Formal critical appraisal was not undertaken, consistent with the mapping purpose of a scoping review, and the evidence was therefore described rather than weighted according to methodological quality. Variation in terminology, interventions and outcomes prevented direct comparison, while the small number of studies and their concentration within three countries and specialist or postgraduate settings limit generalisability. Consequently, the evidence cannot establish the effectiveness of acclimatisation or determine which patients or intervention components may be most likely to benefit.

Future research should clearly distinguish equipment and environmental familiarisation, behavioural preparation or graded exposure, and pharmacological exposure to nitrous oxide/oxygen. Intervention timing, duration, nitrous oxide concentration, personnel, intended purpose and comparator care should be reported explicitly. Adequately powered prospective studies are required using clearly defined and comparable outcomes, including anxiety, cooperation, treatment completion, adverse events, subsequent treatment pathways, resource use and environmental impact. Incorporation of patient-reported outcome measures (PROMs) may also help provide greater insight into children’s and families’ experiences of acclimatisation pathways and inhalation sedation care [33].

## 5. Conclusions

Acclimatisation with inhalation sedation is inconsistently defined and encompasses differing combinations of equipment familiarisation, behavioural preparation and pharmacological exposure. Only one small, underpowered study directly evaluated prior nitrous oxide/oxygen exposure, while the other completed study did not isolate a separate acclimatisation appointment. The available evidence is therefore insufficient to determine whether acclimatisation improves patient or treatment outcomes, or which children might benefit. Neither its routine provision nor its abandonment can be supported on the current evidence. Where used, acclimatisation should remain based on individual assessment and clinical judgement. Future studies require explicit intervention definitions, adequately powered prospective designs, and consistent patient, clinical and service outcomes.

## Figures and Tables

**Table 1:** Characteristics of the three included studies reported across four reports.

Study Report(s)	Evidence Status and Setting	Participants	Intervention and Comparator	Outcomes	Key Results
**Veerkamp et al. (1991)** [24]	Completed randomised comparative clinical study; paediatric dental fear clinic, Netherlands	56 fearful children aged 6–11 years with previous negative dental experiences	Both groups received introductory behavioural procedures. One group also received nitrous oxide during dental treatment; the comparator received behavioural management alone.	Treatment time and number of visits across successive treatment sessions	Mean treatment sessions were 4.8 (SD 1.2) with behavioural management alone and 5.2 (SD 1.5) with additional nitrous oxide; the difference was not statistically significant. Initial familiarisation took longer with nitrous oxide for both dentists: 20 compared with 36 min (*p* = 0.05) and 24 compared with 41 min (*p* = 0.04). Total treatment time was longer with nitrous oxide for one dentist (158 compared with 218 min; *p* = 0.02), but not the other (116 compared with 147 min).
**Al Halabi et al. (2019)** [21] and Kowash et al. (2020) [22]	Two protocol reports describing the same planned single-centre, investigator-blinded randomised parallel-group trial; postgraduate dental hospital, UAE. No outcome data were reported.	Planned recruitment of children aged 5–15 years requiring dental treatment under IHS	Approximately 15 min of nitrous oxide/oxygen exposure during a separate preparatory visit before operative treatment. The comparator received an explanation of IHS without practical exposure.	Planned behavioural and anxiety measures, treatment completion, salivary alpha-amylase and cortisol, and real-time physiological monitoring	No results reported; protocol publications only.
**Palikaraki (2022)** [23]	Completed non-blinded randomised controlled study reported in an unpublished postgraduate thesis; registered as NCT06056492; UK paediatric dental hospital setting	20 children aged 5–15 years, with a mean age of 10.35 years; nitrous oxide/oxygen group *n* = 11 and comparator group *n* = 9	Both groups received a prophylaxis while wearing a nasal hood during assessment. The intervention group received nitrous oxide/oxygen, while the comparator received oxygen alone. Both groups subsequently underwent treatment under IHS.	MCDASf scores, heart rate, skin temperature, treatment completion and patient experience	Mean reduction in MCDASf score from baseline to the end of treatment was 2.55 (SD 8.02) following nitrous oxide/oxygen exposure and 7.22 (SD 5.12) following oxygen-only mask familiarisation. The between-group difference in change was not statistically significant (mean difference −4.68; *p* = 0.148). Treatment was completed by 10 of 11 and 9 of 9 children, respectively (*p* = 1.000).

**Key**: IHS—inhalation sedation; MCDASf—Modified Child Dental Anxiety Scale, faces version; SD—standard deviation.

## Data Availability

The materials and data underpinning the findings of this review can be obtained from the corresponding author upon reasonable request.

## Supplementary Materials

The following supporting information can be downloaded at: https://www.mdpi.com/article/10.3390/dj14100623/s1, Supplementary File S1: Search Strategies.
