# Supplementary material for: Acclimatisation with Inhalation Sedation in Paediatric Dentistry: A Scoping Review
Source: Dent J (Basel). 2026 Sep 30;14(10):623. doi: 10.3390/dj14100623 (PMC13703886; doi:10.3390/dj14100623)
Supplement: Supplementary file 1 [file dentistry-14-00623-s001.zip › dentistry-4515178-supplementary.pdf]

Supplementary File S1: Search Strategies

Ovid Embase

Line	Search strategy
**1**	exp nitrous oxide/ or exp inhalation anesthesia/
**2**	(inhalation sedation or relative analgesia or "gas and air" or happy air or laughing gas or (nitrous oxide and (sedation or anaesthe* or anesthe*))).tw.
**3**	1 or 2
**4**	exp dentistry/ or exp dental care/
**5**	(dentist* or dental).mp.
**6**	4 or 5
**7**	exp acclimatization/
**8**	exp systematic desensitization/
**9**	exp cognitive therapy/
**10**	exp behavior therapy/
**11**	(acclimati?ation or acclimation or systematic desensiti?ation or cbt or cognitive behavior* therap* or cognitive behaviour* therap* or graded exposure or familiari?ation or (prepar* adj2 appointment*) or (trial adj1 run*)).mp.
**12**	(introduc* or acceptance).ti.
**13**	7 or 8 or 9 or 10 or 11 or 12
**14**	exp child/ or exp preschool child/ or exp adolescent/ or exp pediatric dentistry/
**15**	(pediatric or paediatric or child or children).mp.
**16**	14 or 15
**17**	3 and 6 and 13 and 16

Cochrane Library

Line	Search strategy
**#1**	MeSH descriptor: [Nitrous Oxide] explode all trees
**#2**	("inhalation sedation" OR "relative analgesia" OR "gas and air" OR "happy air" OR "laughing gas" OR ("nitrous oxide" AND (sedation OR anaesthe* OR anesthe*))):ti,ab,kw
**#3**	#1 OR #2
**#4**	MeSH descriptor: [Dentistry] explode all trees
**#5**	MeSH descriptor: [Dental Care] explode all trees
**#6**	(dentist* OR dental):ti,ab,kw
**#7**	#4 OR #5 OR #6
**#8**	MeSH descriptor: [Acclimatization] explode all trees
**#9**	MeSH descriptor: [Desensitization, Psychologic] explode all trees
**#10**	MeSH descriptor: [Cognitive Behavioral Therapy] explode all trees
**#11**	MeSH descriptor: [Behavior Therapy] explode all trees
**#12**	(acclimati?ation OR acclimation OR systematic desensiti?ation OR cbt OR (cognitive NEAR/2 behavior* NEAR/2 therap*) OR (cognitive NEAR/2 behaviour* NEAR/2 therap*) OR "graded exposure" OR (behavior* NEAR/1 therap*) OR (behaviour* NEAR/1 therap*) OR familiari?ation OR (prepar* NEAR/2 appointment*) OR (trial NEAR/1 run*)):ti,ab,kw
**#13**	(introduc* OR acceptance):ti
**#14**	#8 OR #9 OR #10 OR #11 OR #12 OR #13
**#15**	MeSH descriptor: [Child] explode all trees
**#16**	MeSH descriptor: [Pediatric Dentistry] explode all trees
**#17**	MeSH descriptor: [Child, Preschool] explode all trees
**#18**	MeSH descriptor: [Dental Care for Children] explode all trees
**#19**	MeSH descriptor: [Adolescent] explode all trees
**#20**	(pediatric OR paediatric OR child OR children):ti,ab,kw
**#21**	#15 OR #16 OR #17 OR #18 OR #19 OR #20
**#22**	#3 AND #7 AND #14 AND #21

Scopus

Line	Search strategy
**#1**	TITLE-ABS-KEY("inhalation sedation" OR "relative analgesia" OR "gas and air" OR "happy air" OR "laughing gas" OR ("nitrous oxide" AND (sedation OR anaesthe* OR anesthe*)))
**#2**	TITLE-ABS-KEY(dentist* OR dental OR dentistry)
**#3**	TITLE-ABS-KEY(acclimati?ation OR acclimation OR (systematic W/1 desensiti?ation) OR cbt OR (cognitive W/2 behavior* W/2 therap*) OR (cognitive W/2 behaviour* W/2 therap*) OR "graded exposure" OR (behavior* W/1 therap*) OR (behaviour* W/1 therap*) OR familiari?ation OR (prepar* W/2 appointment*) OR (trial W/1 run*)) OR TITLE(introduc* OR acceptance)
**#4**	TITLE-ABS-KEY(pediatric OR paediatric OR child OR children OR adolescen*)
**#5**	#1 AND #2 AND #3 AND #4

Web of Science Core Collection

Line	Search strategy
**#1**	TS=("inhalation sedation" OR "relative analgesia" OR "gas and air" OR "happy air" OR "laughing gas" OR ("nitrous oxide" AND (sedation OR anaesthe* OR anesthe*)))
**#2**	TS=(dentist* OR dental OR dentistry)
**#3**	TS=(acclimati?ation OR acclimation OR (systematic NEAR/1 desensiti?ation) OR cbt OR (cognitive NEAR/2 behavior* NEAR/2 therap*) OR (cognitive NEAR/2 behaviour* NEAR/2 therap*) OR "graded exposure" OR (behavior* NEAR/1 therap*) OR (behaviour* NEAR/1 therap*) OR familiari?ation OR (prepar* NEAR/2 appointment*) OR (trial NEAR/1 run*)) OR TI=(introduc* OR acceptance)
**#4**	TS=(pediatric OR paediatric OR child OR children OR adolescen*)
**#5**	#1 AND #2 AND #3 AND #4
